# Dietary Supplementation of Inulin Contributes to the Prevention of Estrogen Receptor-Negative Mammary Cancer by Alteration of Gut Microbial Communities and Epigenetic Regulations

**DOI:** 10.3390/ijms24109015

**Published:** 2023-05-19

**Authors:** Huixin Wu, William J. Van Der Pol, Laura G. Dubois, Casey D. Morrow, Trygve O. Tollefsbol

**Affiliations:** 1 Department of Biology, College of Arts and Science, University of Alabama at Birmingham, Birmingham, AL 35233, USA; huixin3@uab.edu; 2 Department of Microbiology, Heersink School of Medicine, University of Alabama at Birmingham, Birmingham, AL 35205, USA; 3 Center for Clinical and Translational Science, University of Alabama at Birmingham, Birmingham, AL 35233, USA; 4 Proteomics and Metabolomics Core Facility, Duke University Medical Center, Durham, NC 27701, USA; 5 Department of Cell, Departmental & Integrative Biology, Heersink School of Medicine, University of Alabama at Birmingham, Birmingham, AL 35294, USA; 6 O’Neal Comprehensive Cancer Center, Heersink School of Medicine, University of Alabama at Birmingham, Birmingham, AL 35294, USA; 7 Integrative Center of Aging Research, University of Alabama at Birmingham, Birmingham, AL 35294, USA; 8 Nutrition Obesity Research Center, University of Alabama at Birmingham, Birmingham, AL 35294, USA; 9 Comprehensive Diabetes Center, Heersink School of Medicine, University of Alabama at Birmingham, Birmingham, AL 35294, USA; 10 University Wide Microbiome Center, University of Alabama at Birmingham, Birmingham, AL 35294, USA

**Keywords:** prebiotics, inulin, breast cancer, gut microbiome, epigenetics regulations

## Abstract

Breast cancer (BC) is among the most frequently diagnosed malignant cancers in women in the United States. Diet and nutrition supplementation are closely related to BC onset and progression, and inulin is commercially available as a health supplement to improve gut health. However, little is known with respect to inulin intake for BC prevention. We investigated the effect of an inulin-supplemented diet on the prevention of estrogen receptor-negative mammary carcinoma in a transgenic mouse model. Plasma short-chain fatty acids were measured, the gut microbial composition was analyzed, and the expression of proteins related to cell cycle and epigenetics-related genes was measured. Inulin supplementation greatly inhibited tumor growth and significantly delayed tumor latency. The mice that consumed inulin had a distinct microbiome and higher diversity of gut microbial composition compared to the control. The concentration of propionic acid in plasma was significantly higher in the inulin-supplemented group. The protein expression of epigenetic-modulating histone deacetylase 2 (Hdac2), Hdac8, and DNA methyltransferase 3b decreased. The protein expression of factors related to tumor cell proliferation and survival, such as Akt, phospho-PI3K, and NF-kB, also decreased with inulin administration. Furthermore, sodium propionate showed BC prevention effect in vivo through epigenetic regulations. These studies suggest that modulating microbial composition through inulin consumption may be a promising strategy for BC prevention.

## 1. Introduction

Breast cancer (BC) is one of the most frequently diagnosed cancers in women worldwide, and its incidence and mortality rates have been increasing over the past decades [1]. Risk factors of BC include female sex, age, genetic mutations, menstrual period, environmental pollution, and family history [1,2]. About 20% of BC patients are estrogen receptor-negative (ER-negative) and approximately 15% are triple-negative BC, suggesting the importance of ER-negative cancer prevention [3]. While hormone receptor-positive patients have more choices of treatment such as aromatase inhibitors and selective estrogen receptor modulators, ER-negative BC patients have fewer options, and the primary treatments are surgery and chemotherapies [4].

The increasing diagnosis and high mortality rates bring importance to cancer prevention. It has been estimated that a great number of cancer deaths can be prevented by healthy diet consisting in part of fruits, vegetables, whole grains, and nuts [5]. A healthy diet prevents BC development by affecting gut microbiota, metabolism, oxidation, and host immune functions. As an important component of a healthy diet, fiber has been associated with reduced risk of BC, in part because dietary fiber can decrease the circulating estrogen. Soluble and insoluble fiber was found to be inversely associated with risk of both ER-positive and ER-negative BC [6]. The mechanism of dietary fiber in cancer prevention is mediated by gut microbiota. As our ‘forgotten organ’, gut microbiota closely regulates the gut homeostasis in hosts. Both intestinal and mammary microbiomes were hypothesized to be associated with breast cancer risk [7]. Dysbiosis of microbiota that harbor protumoral bacterial species can promote tumorigenesis such as gastrointestinal cancers by a variety of mechanisms including activation of inflammatory genes, producing reactive oxygen species that lead to DNA damage, and increasing toxic nitrogenous compounds in the gut [8,9]. Gut microbiota digest soluble and insoluble dietary fiber and other nutrients to harvest energy, fermenting fiber to generate health-benefiting metabolites such as short-chain fatty acids (SCFAs), and more importantly interact with host immunity [7,10].

A variety of types of dietary fiber, including polysaccharides and oligosaccharides, can modulate gut microbiota, and the metabolic products that are generated by bacteria in the GI tract have positive and beneficial impact on the host [10]. Inulin is a commercially available prebiotic among soluble fiber and is found in a wide variety of foods such as whole wheat, garlic, onions, artichokes, and asparagus. The daily intake is 1–10 g/day/person by estimation [11]. Unable to be digested or absorbed by the stomach, inulin can be metabolized by bacteria in the GI tract to improve the growth of several butyrate-producing bacteria, including *Roseburia intestinalis*, *Enbacterium rectale*, and *Anserostiples caccae*, as well as health-promoting probiotics *Bifidobacterium bifidum* and *Faecalibacterium prausnitzii* [12,13,14].

The cancer prevention effect of prebiotics including inulin has been hypothesized to be mediated by inflammation, bacteria-host immunity interactions, estrogen metabolism, and epigenetic alterations [15,16]. Dietary inulin has been shown to promote the proliferation of beneficial microbiota and was able to increase SCFAs, bile acids, and glycolytic metabolites to reduce neuroinflammation in an Alzheimer’s disease mouse model [17]. A colorectal cancer mouse model treated with inulin showed increased CD45+ cells including CD4+ and CD8+ T cells and plasmacytoid CD8+ T cells [18]. In addition to the well-known role of host-immunity in cancer prevention, epigenetics is also an important factor because it greatly impacts posttranslational changes during tumor initiation and progression [19]. Gut microbiota have been known to induce epigenetic changes including DNA methylation and histone modifications in metabolic diseases mediated by metabolites such as SCFAs, folates, and trimethylamine-N-oxide generated by gut microbiota [20]. However, little is known about the epigenetics influence of the prebiotic inulin on BC prevention.

Dietary intervention is a promising strategy to reverse dysregulation of epigenetic modifications that are associated with tumorigenesis [21]. The anticancer effect of bioactive compounds such as phytochemicals are mediated by epigenetic regulations such as inhibiting DNA methyltransferases (DNMTs), inhibiting histone deacetylase (HDACs), and modulating microRNA [22]. In addition, SCFAs have been found to induce epigenetic alterations. For examples, butyrate and propionate can inhibit HDACs which regulate tumor-related genes and activate tumor-suppressing genes [20]. Therefore, we hypothesize that dietary fiber inulin can stimulate the growth of health-benefiting bacteria that increase the level of circulating metabolites such as SCFAs to affect key epigenetic regulators in tumors and contribute to BC prevention. Discovering naturally occurring HDACs and DNMTs inhibitors that present promising anticancer activities will facilitate the development of cancer prevention strategies and adjuvant BC therapies that can be applied to multiple BC subtypes.

## 2. Results

### 2.1. Inulin-Supplemented Diets Were Effective in Preventing ER-Negative Breast Tumor Development in Mice

**Figure 1:**
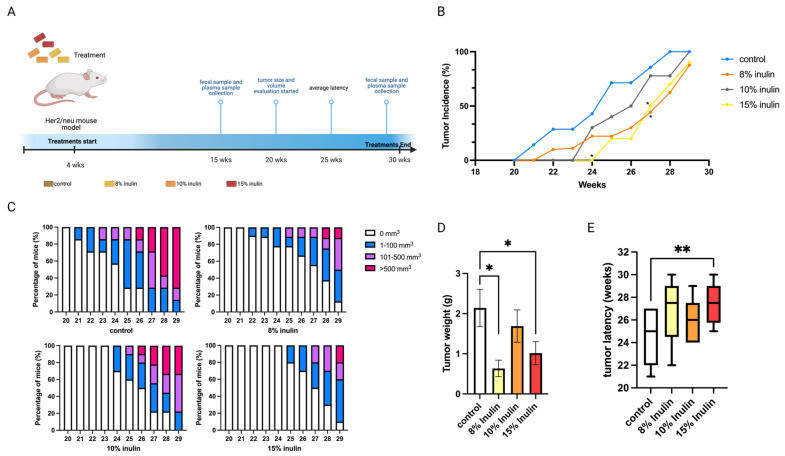
Tumor growth in mice with inulin dietary treatment. (**A**) Schematic representation of experimental design for inulin dietary treatment. Female Her2/neu transgenic mice were administered 8%, 10%, or 15% (*w*/*w*%) of inulin-supplemented diets from weaning to the termination of the experiment. Tumor growth was monitored weekly. (**B**) Tumor incidence. (**C**) Tumor growth volume. (**D**) Tumor weight measured at the termination point. (**E**) Tumor latency. Columns, mean; Bars, SEM; *, *p* < 0.05; **, *p* < 0.01, significantly different from the control group.

The Her2/neu transgenic mouse model was used in this study because the overexpression of the *Erbb2* gene drives the development of ER-negative mammary tumors, and this mouse model is suitable for studying human breast cancer. Inulin treatment concentrations were determined by previous publications whereby 8% inulin (40 g fiber/day) was proven to be physiologically reasonable for treating human patients, and 15% inulin was the high concentration that has been used to treat mice [17,18]. This study explored the cancer prevention effect of 8%, 10%, and 15% inulin-supplemented diets (IN) on ER-negative mammary tumor development. The treatment and sample collection time points are illustrated in (Figure 1A).

All inulin-supplemented diets decreased the tumor incidence compared to the control and 8% and 15% significantly decreased the tumor incidence at 27 weeks of age (Figure 1B). At 8% and 15%, IN in the diet also prominently suppressed tumor volume and significantly decreased tumor weight (Figure 1C,D) while 15% IN significantly delayed tumor latency (Figure 1E). Notably, 15% IN delayed tumor onset and had longer tumor latency and the 8% IN had a stronger inhibiting effect on tumor weight and volume.

### 2.2. Impact of Inulin-Supplemented Diets on Gut Microbial Composition before and after the Tumor Development

As dietary fiber and prebiotics, inulin can promote the growth of health-benefiting bacteria. Therefore, the gut microbial composition was investigated in the Her2/neu transgenic mouse model. A longitudinal study that compared the gut microbial composition of each treatment group before and after tumor development was also performed. The Her2/neu transgenic mouse model develops ER-negative mammary tumors at around 20 weeks of age. Our study investigated the gut microbial changes induced by inulin-supplemented diets before the onset of tumor (15 weeks of age) and after the onset of tumor (25 weeks of age) (Figure 1A). Since the cancer prevention effect was more profound in the 8% IN and 15% IN groups (Figure 1B–E), the gut microbial composition was evaluated in the control, 8% IN, and 15% IN group. Fecal samples were collected at 15 and 25 weeks of age for the gut microbial analysis.

#### 2.2.1. Impact of Inulin Diets on Gut Microbial Composition before the Onset of Tumor

**Figure 2:**
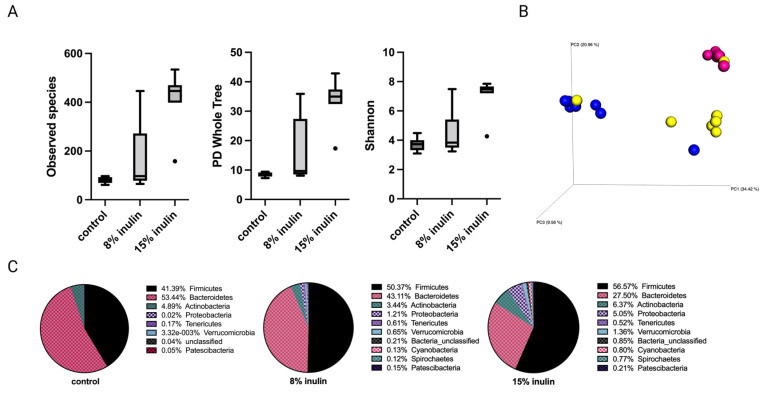
Gut microbial composition changes before tumor development. There was a distinct difference in microbial composition between control and two inulin-treated groups. (**A**) alpha-diversity; observed species, PD whole tree, and Shannon diversity (**B**) 3D-PCoA plot (Bray Curtis) showed distinct clustering of control (blue) and 8% IN (yellow) or 15% IN (red). (**C**) Phylum level changes in microbial abundance before the tumor development.

The alpha diversity displayed as observed species, PD whole tree, and Shannon diversity showed higher diversity of gut microbial composition in the 15% IN group (Figure 2A). The Bray Curtis beta diversity was displayed in the 3D Principal Coordinates Analysis (PCoA) plot (Figure 2B). Distinct clustering of the individual samples in each group was observed. The statistial significance was further validated: Bray Curtis 8% IN (*p* = 0.001), 15% IN (*p* = 0.001); Weighted unifrac 8% IN (*p* = 0.007), 15% IN (*p* = 0.002). The top 10 abundant phyla of all groups are represented in the pie charts. The relative abundance of Bacteroidetes decreased in both treatment groups while the relative abundance of Firmicutes increased. The 15% IN group had higher relative abundance of diverse phyla compared to the control (Figure 2C). These results indicate the inulin-supplemented diets groups increased microbial diversity and harbored distinct microbial composition compared to the control group before the onset of tumor.

Comparison of the relative abundance of microbes of mice gut microbial composition between the different groups was done using Kruskal-Wallis. One-way analysis of variance significance difference in bacterial communities was evaluated after false-discovery rate (FDR) correction. The 8% IN group had no significantly different bacterial community compared to control (Table S1). The 15% IN group had numerous bacterial taxa identified at genus and species levels. The relative abundance of Lacobacillus murinus, Tissierellia bacterium S7-1-4, Balutia, Faecalibaterium, Akkermansia, and Prevotella increased, while Romboutsia, Bifidobacterium, and Muribaculaceae decreased (Table S2). Overall, the high inulin-supplemented diet significantly increased the relative abundance of health-benefiting bacteria compared to the control before tumor development.

#### 2.2.2. Impact of Inulin Diets on Gut Microbial Composition after the Onset of Tumor

**Figure 3:**
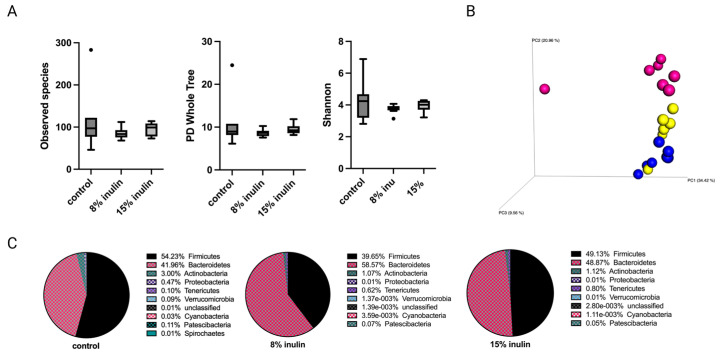
Gut microbial composition changes after tumor development. There was a distinct difference in microbial composition between control and two inulin-treated groups. (**A**) Alpha-diversity; Observed species, PD whole tree, and Shannon diversity (**B**) 3D-PCoA plot (Bray Curtis) showed distinct clustering of control (blue) and 8% IN (yellow) or 15% IN (red). (**C**) Phylum level changes in microbial abundance after the tumor development.

Tumor development has a systemic impact on gut microbiota and whole-body metabolism and immunity [23,24,25]. Therefore, gut microbial composition between groups was investigated after tumor development at the average tumor latency of the Her2/neu mouse model. The alpha diversity was displayed as observed species, PD whole tree, and Shannon diversity. The alpha diversity of gut microbial composition in both inulin-treated groups were not signifcantly different from the control group (Figure 3A). The Bray Curtis beta diversity displayed in the PCoA plot showed distinct clustering of individual samples in each group (Figure 3B). The statistical significances were further analyzed: Bray Curtis 8% IN (*p* = 0.001), 15% IN (*p* = 0.001); weighted unifrac 8% IN (*p* = 0.073), 15% IN (*p* = 0.043). The top 10 abundant phyla in pie charts showed a decrease of Firmicutes to Bacteroidetes ratio in both inulin-treated groups compared to the control (Figure 3C). These results indicate that the inulin-induced gut microbial composition changes before tumor formation were reversed by tumor development.

The relative abundance of bacterial taxonomies of gut microbial composition was analyzed by Kruskal-Wallis with FDR correction. The 8% IN group had no significant difference in bacterial community compared to control (Table S3). The 15% IN group had bacterial taxa identified at the Family level. The relative abundance of Clostridiaceae and Peptostreptococcaceae decreased significanly in the 15% IN group compared to the control group (Table S4). Though the high inulin-group had significantly different bacterial taxa, bacterial taxonomies that had significantly different relative abundance between inulin-treated groups and control groups after tumor development (*n* = 3) was not as abundant as that before tumor development (*n* = 89) (Tables S2 and S4).

Longitudinal changes of bacterial communities within the same group over time were also investigated. Within each treatment group, the relative abundance of bacterial communities was compared between two time points: before and after the onset of tumor. The control group and 8% IN group did not show significantly different microbial compositions over time (Tables S5 and S6). The 15% IN group showed numerous significantly different bacterial taxa after the onset of tumor (25 weeks of age) compared to before the onset of tumor (15 weeks of age). Kruskal-Wallis with FDR correction identified significant decreases of Prevotella, Staphylococcus aureus, Anaerococcus, Tissierellia bacterium S7-1-4, Blautia, Varibaculum, Lactobacillus murinus, Faecalibacterium, Akkermansia, Lactobacillus reuteri, Blautia, and Faecalibaculum while significant increases of Muribaculaceae, and Ruminococcaceae UCG-014 were observed (Table S7). Higher abundance of SCFA-producing bacteria Faecalibacterium and Akkermansia were found in inulin groups before the tumor onset, but their population decreased after the tumor onset [20]. Overall, these results indicate BC mammary cancer had a negative impact on several health-benefiting gut microbial modifications of the inulin-supplemented diets.

### 2.3. Analysis of the SCFAs Profile in Mice on the Inulin-Supplemented Diets

**Figure 4:**
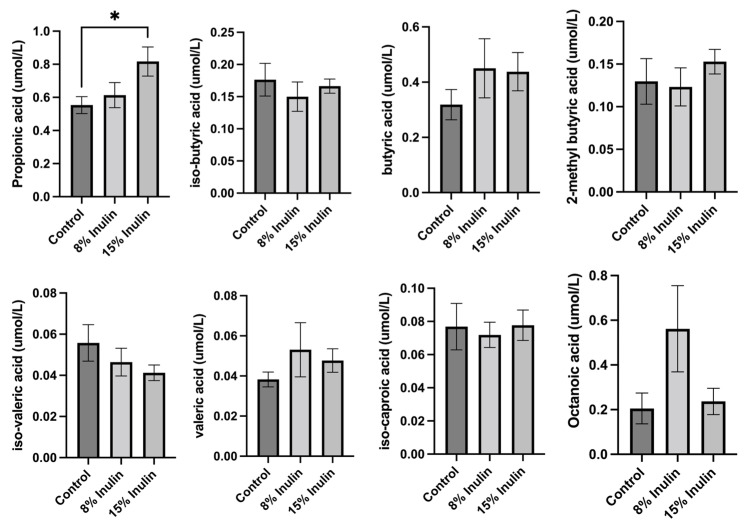
Analysis of plasma SCFAs levels in 8% IN, 15% IN, and control groups. Concentrations of SCFAs propionic acid, isobutyric acid, butyric acid, 2-methyl butyric acid, isovaleric acid, and valeric acid in inulin dietary treatment mice versus the control group are shown. Concentrations of medium-chain fatty acids isocaproic acid and octanoic acid in inulin dietary treatment mice versus the control group are also shown. Columns, mean; bars, SEM; *, *p* < 0.05, significantly different from the control group.

The changes induced in the gut microbial communities by inulin-supplemented dietary groups were hypothesized to have an impact on ER-negative mammary tumors mediated by circulating metabolites because previous findings have shown that bacterial metabolites can be transferred to distant organs through circulation [10,26]. Therefore, we hypothesized that gut microbes can ferment dietary fiber to produce SCFAs that can travel to distal organs by the circulatory system. SCFAs are also known as epigenetics regulators that potentially contribute to BC prevention [27]. Therefore, SCFAs profiles in the plasma were measured and compared between treatment groups. Propionic acid, isobutyric acid, butyric acid, 2-methyl butyric acid, isovaleric acid, valeric acid, isocaproic acid, and octanoic acid levels were measured by LC-MS/MS. The differences of metabolites were compared between inulin-treated groups to the control group. Butyric acid and valeric acid levels increased, but not significantly, in two inulin-treated groups. Propionic acid level increased significantly in 15% IN group (Figure 4).

### 2.4. Inulin-Supplemented Diets Result in Changes in Protein Expression of Tumor-Related Genes and Epigenetic Regulators

**Figure 5:**
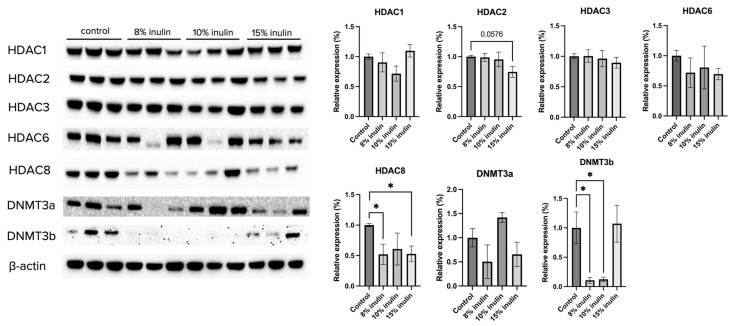
Protein expression changes of key epigenetics modification-related factors in the mammary tumor of the inulin-treated and control groups. Western blotting was performed to measure the protein expression changes of Hdac 1, Hdac 2, Hdac 3, Hdac 6, Hdac 8, Dnmt3a, and Dnmt3b in mammary tumor samples of the inulin-treated groups and control group. β-actin was used as a loading control. *n* = 3. Columns, mean; bars, SEM; *, *p* < 0.05, significantly different from the control group.

Gut microbial composition and circulating SCFAs are hypothesized to affect the ER-negative mammary tumors in the Her2/neu mouse model. To explore the molecular mechanisms by which inulin-supplemented dietary treatment suppressed mammary tumor growth, the protein expression of several epigenetic-modulatory enzymes, including HDAC1, HDAC2, HDAC3, HDAC6, HDAC8, DNMT3a, and DNMT3b, were evaluated. The results indicate that 15% IN greatly but not significantly reduced the expression of HDAC2 (*p* = 0.0576). Both 8% IN and 15% IN significantly reduced the expression of HDAC8, as well as expression of DNMT3b (Figure 5). The results suggest important roles of the inulin-supplemented diets in inhibition of key epigenetic regulators in mammary tumors.

**Figure 6:**
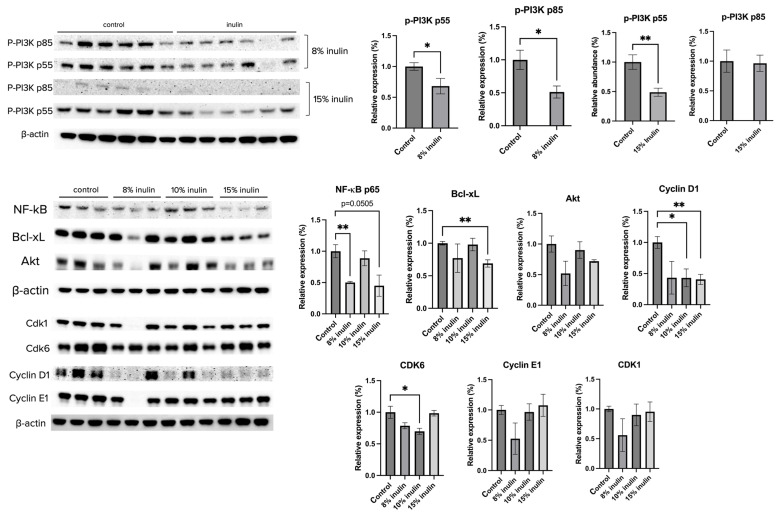
Protein expression changes of key factors in the cell cycle and Akt/PI3K pathways and downstream targets in the mammary tumor of the inulin-treated and control groups. Western blotting was performed to measure the protein expression changes of p-PI3K p55, p-PI3K p85, Akt, NK-kB, Bcl-xL, Cyclin D1, Cyclin E1, CDK1, and CDK6 in mammary tumor samples of the inulin-treated groups and control group. β-actin was used as a loading control. *n* = 3 or 6 as depicted in the graph. Columns, mean; bars, SEM; *, *p* < 0.05; **, *p* < 0.01, significantly different from the control group.

The protein expression of key factors of the PI3K/Akt pathway and the cell cycle arrest pathway were investigated. Administration of 8% IN significantly decreased the expression of P-PI3K p55 and p85, while 15% IN significantly decreased the expression of p-PI3K p55 (Figure 6). The protein expression of downstream factors that regulate cell survival and apotosis, NF-kB p65 and Bcl-xL, respectively, were significantly decreased in both inulin-supplemented dietary groups. Cell cycle regulators, such as Cyclin D1 and CDK6, were significantly decreased in inulin-treated groups (Figure 6). The results help illuminate potential mechanisms of inulin diets in ER-negative BC prevention.

### 2.5. Inulin-Supplemented Diets Induced Changes in Histone Deacetylase, DNA Methyltransferase, and Histone Acetyltransferase

**Figure 7:**
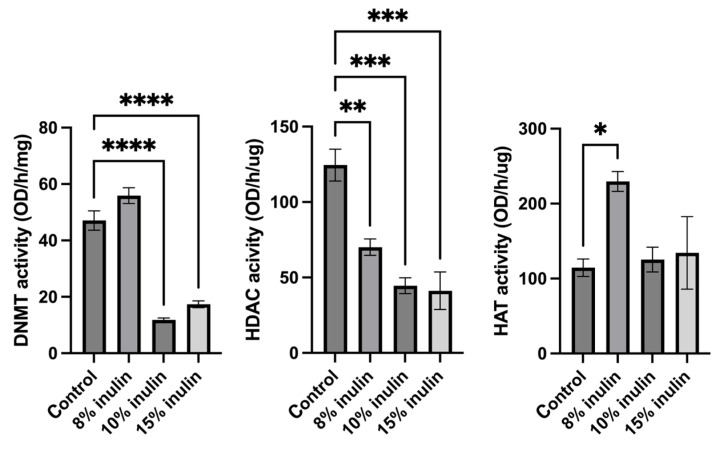
Histone deacetylase (HDAC) activity, DNA methyltransferase (DNMT) activity, and histone acetyltransferase (HAT) activity levels in the ER-negative mammary tumors. Triplicates were randomly selected mouse mammary tumors from each treatment group. Columns, mean; bars, SEM; *, *p* < 0.05; **, *p*< 0.01; ***, *p* < 0.001; ****, *p* < 0.0001, significantly different from the control group.

Epigenetic mechanisms were further explored by determining enzymatic changes of epigenetic modifiers, including HDACs, DNMTs, and HAT, in the mammary tumors. Nuclear protein extracted from tumor samples were used for enzymatic activity assays. All three inulin-treated groups significantly decreased HDACs activity. Notably, 10% IN and 15% IN groups significantly decreased DNMTs activity and 8% IN group increased the HAT activity compared to the control group (Figure 7). The results indicate that inulin-supplemented diets inhibited the enzymatic activities of key epigenetic-modulatory enzymes.

### 2.6. Sodium Propionate Inhibited Cell Viability and Proliferation in MDA-MB-231, MDA-MB-157, MCF-7, and T47D Human BC Cells

**Figure 8:**
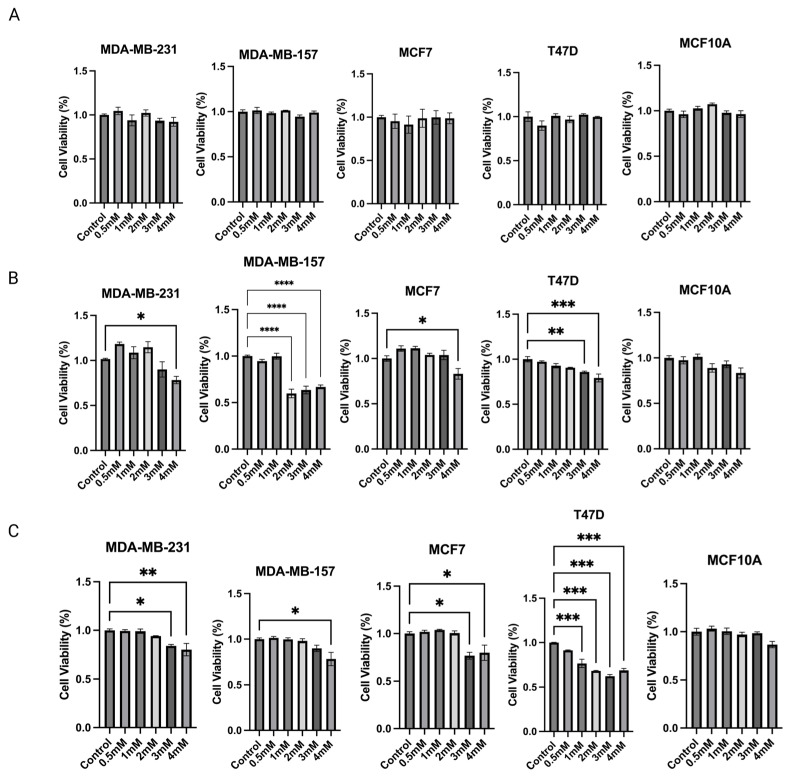
MTT assay was performed for the cell viability of sodium propionate (SP)-treated groups compared to the control group. Inhibition of cell viability in MDA-MB-231, MDA-MB-157, MCF-7, and T47D human breast cancer cells after treatment with 0, 0.5, 1, 2, 3, 4 mM SP for (**A**) 24 h, (**B**) 48 h, (**C**) 72 h. MCF10A human mammary epithelial cells were used as the control cell line to determine the toxicity of SP treatments. Columns, mean; bars, SEM; *, *p* < 0.05; **, *p* < 0.01; ***, *p* < 0.001; ****, *p* < 0.0001, significantly different from the control group.

Based on the above findings, propionic acid was identified as a SCFA, epigenetic regulator, that increased significantly in 15% IN group. Changes in protein expressions and enzymatic activities of epigenetic modulators further indicate that propionic acid potentially mediates the gut microbial composition modifications and epigenetics regulations in tumors. Therefore, the cell toxicity effect of propionic acid was tested on human BC cell lines. To determine the anticarcinogenic effect of sodium propionate (SP), four human BC cell lines, MDA-MB-231, MDA-MB-157, MCF-7, and T47D, were treated with SP (0.5, 1, 2, 3, 4 mM) for 24 h, 48 h, or 72 h in a dose- and time-dependent manner when compared with the DMSO-treated control group of the same cell lines (Figure 8A–C). SP treatment exhibited a dose- and time-dependent inhibitory effect on cancer cell lines except for MDA-MB-157. SP at 3 mM and 4 mM significantly inhibited cancerous cell viability in the 72 h treatment period. MCF10A was used as a control noncancerous cell line to test for potential cell toxicity of SP (Figure 8C). SP at 0.5–4 mM exhibited little to no apparent reduction in cell viability, suggesting the 4 mM SP for 72 h treatment is viable for further analysis.

### 2.7. Sodium Propionate Inhibited HDAC and DNMT Enzymes Activities in MDA-MB-231, MDA-MB-157, and T47D Human BC Cells

**Figure 9:**
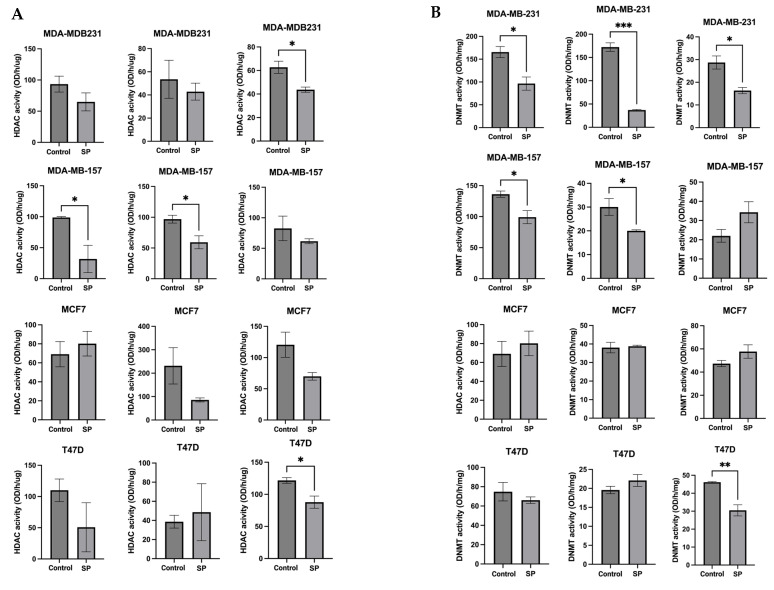
Histone deacetylase (HDAC) activity and DNA methyltransferase (DNMT) activity levels in the human breast cancer cell lines. (**A**) Histone deacetylase activity of MDA-MB-231, MDA-MB-157, MCF-7, and T47D cell lines treated with 4 mM SP for 72 h. (**B**) DNA methyltransferase activity of MDA-MB-231, MDA-MB-157, MCF-7, and T47D cell lines treated with 4 mM SP for 72 h. Columns, mean; bars, SEM; *, *p* < 0.05; **, *p* < 0.01; ***, *p* < 0.001, significantly different from the control group.

To further explore the epigenetic mechanisms, the enzymatic activities of epigenetic modifiers HDACs and DNMTs were measured in human BC cell lines treated with 4 mM SP for 24 h, 48 h, and 72 h. SP treatment significantly reduced HDACs activity in the MDA-MB-157 cell line after 48 h treatment. SP at 4 mM treatment significantly reduced HDACs activity in MDA-MB-157 after 48 h treatment and MDA-MB-23 and T47D cell lines after 72 h treatment (Figure 9A). DNMTs activity was inhibited in MDA-MB-231 cell lines after 24 h, 48 h, and 72 h of treatment. MDA-MB-157 cells had significantly reduced DNMT activity after 24 h and 48 h of treatment. T47D cells showed great inhibition of DNMT activity after 72 h of treatment (Figure 9B). There results indicate the inhibitory effect of SP on key epigenetic-modulatory enzymes in human BC cell lines.

### 2.8. Sodium Propionate Treatment Delayed ER-Negative Breast Tumor Development in Mice

**Figure 10:**
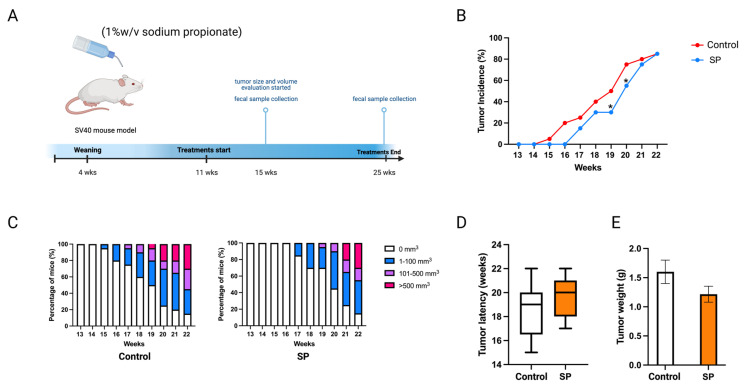
Tumor growth in mice with 1 *w*/*v*% SP-added drinking water treatment. (**A**) Schematic representation of experimental design for SP treatment. Female C3(1)-TAg transgenic mice were administered 1% (*w*/*v*%) of SP-added drinking water from 11 weeks of age to the termination of the experiment. Tumor growth was monitored weekly. (**B**) Tumor incidence. (**C**) Tumor growth volume. (**D**) Tumor latency. (**E**) Tumor weight measured at the termination point. Columns, mean; bars, SEM; *, *p* < 0.05; significantly different from the control group.

Sodium propionate inhibited human BC cell growth and the enyzme activities of epigenetic modifiers. The cancer prevention effect of sodium propionate was further evaluated in vivo. C3(1)-TAg transgenic mouse model was used as it spontaneously develops ER-negative mammary tumors at 15 weeks of age. This transgenic model does not over-express Erbb2; therefore, it can potentially serve as a model for not only ER-negative but also triple-negative BC studies [28,29]. Moreover, this mouse model has a much shorter latency than the Her2/neu model which avoids the potential toxicity of SP to the mice. The concentration was determined by previous studies; 1 *w*/*v*% or 2 *w*/*v*% of SP in drinking water has been used to treat mouse models [30,31]. In this study, 1 *w*/*v*% sodium propionate was added to the drinking water and water was replaced every three days (Figure 10A). The SP-treated group significantly decreased tumor incidence at 19 and 20 weeks of age compared to the control group (Figure 10B). SP treatment greatly suppressed tumor volume (Figure 10C). The treatment also delayed tumor development and reduced tumor weight, though not significantly (Figure 10D,E). Overall, 1% SP in drinking water significantly decreased tumor incidence and delayed tumor development.

### 2.9. Sodium Propionate Treatment Results in Changes in Protein Expression of Tumor-Related Genes and Epigenetic Regulators and Inhibited HDAC Enzyme Activity

**Figure 11:**
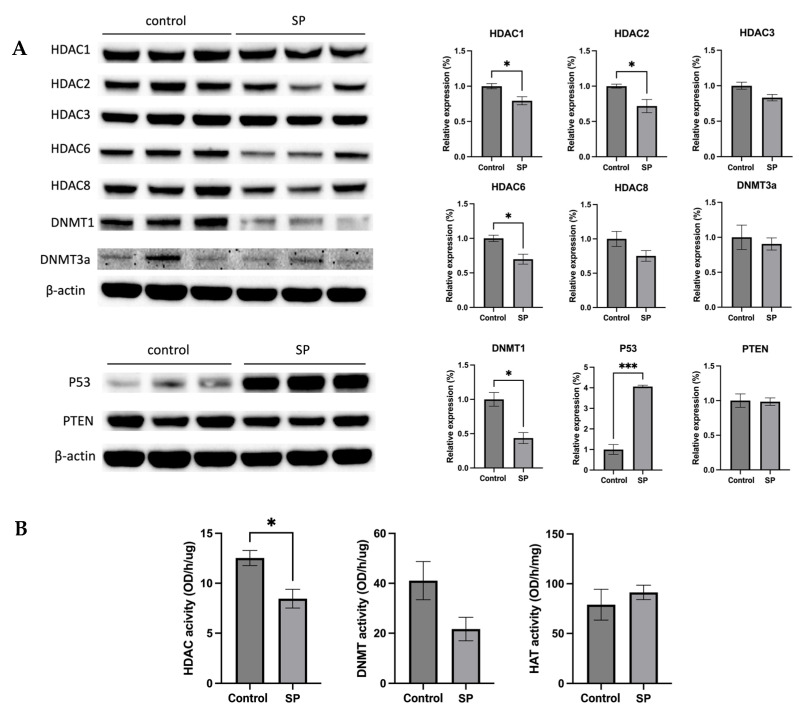
Protein expression and enzymatic activities changes of epigenetics modification-related genes in the mammary tumor of the sodium propionate-treated group and control group. (**A**) The protein expression changes of Hdac 1, Hdac 2, Hdac 3, Hdac 6, Hdac 8, Dnmt3a, and Dnmt1 in mammary tumors by Western blotting. β-actin was used as a loading control. (**B**) HDAC activity, DNMT activity, and HAT activity levels in the mammary tumors. Triplicates were randomly selected from each of the treatment groups. *n* = 3. Columns, mean; bars, SEM; *, *p* < 0.05; ***, *p* < 0.001, significantly different from the control group.

The anticancer effect of SP treatment was studied in vitro and in vivo. Next, the molecular mechanism by which 1 *w*/*v*% sodium propionate treatment suppressed mammary tumor growth in the SV40 mouse model was further investigated. The protein expression of epigenetic regulators, including HDAC1, HDAC2, HDAC3, HDAC6, HDAC8, DNMT3a, and DNMT1, were evaluated. The treatment significantly reduced the expression of HDAC1, HDAC2, HDAC6, and DNMT1 (Figure 11A). The protein expression of tumor suppressors P53 and PTEN were also evaluated, and the results show that SP treatment significantly increased the expression of P53 (Figure 11A). The results suggest that SP treatment induced protective epigenetics changes and induced the expression of tumor suppressors in the mammary tumors. Moreover, the enzymatic activities of epigenetic modifiers HDACs, DNMTs, and HAT were measured in mammary tumors. SP treatment significantly reduced HDAC enzymatic activity and greatly suppressed DNMT enzymatic activity, though not significantly (Figure 11B). Overall, the anticancer effect of in vivo SP treatment is in part mediated by elevated protective epigenetic regulations.

## 3. Discussion

The widely used existing BC treatments include chemotherapy followed by hormonal therapy or hormonal therapy alone depending on the age of population [32]. Developing cancer prevention and treatment for ER-negative BC patients who do not benefit as much from the hormonal therapy is needed. Dietary interventions that can improve gut health or induce protective epigenetics modifications have been studied as cancer prevention strategies in the past decade. As a well-known prebiotic, inulin has been used for treating and preventing digestive problems, weight loss, and constipation because it helps increase the minerals absorption from food, promotes the growth of health-benefiting bacteria, and supports a healthy immune system according to previous studies [33,34]. Our investigation explored the potential mechanisms, gut microbiota regulation, and epigenetics modifications of inulin dietary intervention in BC prevention. We investigated the effect and mechanism of inulin-supplemented dietary treatment on ER-negative BC mouse model. The anticancer effect of inulin (IN) treatment was evaluated by tumor incidence, tumor latency, and tumor weight. The 8% IN and 15% IN groups but not 10% IN group exhibited profound anticancer effects based on the evaluation, indicating that the anticancer effect was not dose-dependent. Inulin supplementation has been found to consistently impact the bacterial population of *Bifidobacterium*, *Anaerostipes*, and *Bilophila* [35]. Gut microbial communities can metabolize carbohydrates to release energy, gas, and fermented byproducts to the host [36]. Higher concentration of the inulin treatment can potentially induce greater changes in the gut microbial composition that digest carbohydrate, protein, and bile acid to affect the metabolite and energy absorption of the host, therefore having an impact on the cancer prevention outcome.

The changes in gut microbial community composition were compared between treatment groups to the control group at two time points, before and after the tumor onset. We investigated 8% IN and 15% IN groups because they represent low and high concentrations of inulin treatments, respectively, and because of their profound anticancer effects. Increased microbiome diversity and distinct microbial composition were observed in both inulin-treated groups before the onset of tumor compared to the control, suggesting the inulin-supplemented diets induced prominent changes in the gut microbial composition in ER-negative mouse model. At the phylum level, a higher Firmicutes to Bacteroidetes ratio was observed in treatment groups. However, after the onset of tumor, the microbiome alpha diversity was not significantly different between groups while the beta diversity remained significant. Interestingly, the Firmicutes to Bacteroidetes (F/B) ratio decreased relative to the control group. The F/B ratio has been found to increase in breast tumor compared to normal breast tissue and malignant BC compared to benign disease [37,38]. A decreased F/B ratio in the inulin-treated groups indicates the treatment reversed the gut microbiota changes associated with BC.

Analysis of microbial composition showed significant differences between treatment groups before and after the onset of tumor. Among the numerous bacterial genus and species identified, the increased population of *Lacobacillus murinus*, *Balutia*, *Faecalibaterium*, *Akkermansia*, and *Prevotella* are highlighted in Table S2. As a predominant *Lactobacillus* species in rat gut, *Lactobacillus murinus* can alleviate intestinal reperfusion injury to rescue the high mortality in rats [39,40]. Anaerobic bacteria *Blautia* are found in the feces and intestines of mammals and have probiotic characteristics [41]. *Faecalibacterium* also increased in inulin-treated groups. Under this genus, the species *Faecalibacterium prausnitzii* is a well-known next-generation probiotic with protective effect on human intestinal health [42]. In addition, *Faecalibacterium spp.* is one of the predominant butyrate producers [20]. *Akkermansia* is also a next generation beneficial strain for improving gut health and is known to be positively correlated with IFN-gamma and negatively correlated with immunosuppressive cytokines, thus potentially used to enhance immune response [43,44]. *Akkermansia spp.* is known to produce butyrate and propionate [20]. Under the genus *Prevotella*, the species *Prevotella copri* was found to be associated with high fiber non-Western diets and is also a next generation probiotic [45]. Among the decreased population of bacteria, the decrease of Bifidobacterium in the inulin-treated groups was inconsistent to previous findings [46]. Microbial composition analysis showed increased populations of several health-benefiting bacterial species in inulin-treatment group 15% IN. However, the taxonomy analysis after the onset of tumor had few statistically different bacterial genus or species between treatment groups, indicating that tumor development shaped the gut microbial composition and counteracts the changes brought by inulin diets (Table S4). Furthermore, longitudinal studies were performed to compare the changes of microbial composition within each group before and after the onset of tumor. The 15% IN group exhibited greater changes of gut microbial composition over time (Table S7). At the average tumor latency time point, the populations of several health-benefiting bacteria decreased significantly. Bacteria that have probiotics characteristics, such as *Prevotella*, *Blautia*, *Lactobacillus murinus*, *Faecalibacterium*, *Akkermansia*, *Lactobacillus reuteri*, and *Faecalibaculum,* all decreased compared to before the onset of tumor time point, suggesting that the tumor development weakened the gut microbiome changes induced by inulin treatment. Interestingly, the population of *Muribaculaceae*, a propionate-producer, significantly increased [47].

We found that our inulin treatments shaped the gut microbiome toward health-benefiting populations of bacteria, although the effect was weakened by the onset of tumor. Gut microbiome changes occurring in the GI tract can have an impact on distant organs and tumorigenesis mediated by host immunity and circulating metabolites. The plasma SCFAs were measured in the plasma samples. Metabolites produced by gut bacteria such as butyrate, propionate, and acetate (acetate level was undetectable due to lower than the Limit of Quantitation) were analyzed because SCFAs are well-known epigenetics regulators. Butyrate is a known HDAC inhibitor and can induce apoptosis by increasing reactive oxygen species [48,49]. Sodium propionate was found to be an HDACs inhibitor [50]. Isobutyrate, valerate, and isovalerate, as well as medium-chain fatty acids caproic acid (caproic acid level was undetectable due to lower than the Limit of Quantitation), isocaproic acid, and octanoic acid were also analyzed. Medium chain fatty acids were found to promote histone acetylation to restore the metabolism-related genes [51]. The epigenetic regulating characteristics of SCFAs and increased plasma propionic acid level by inulin treatment led to the hypothesis that propionate can potentially induce epigenetic changes in the mammary tumor site in mice.

Epigenetic regulation in tumorigenesis was investigated by measuring the protein expression and enzymatic activities of key epigenetic regulators. Histone acetylation unwinds the chromatin architecture for basal transcription factors and RNA Polymerase II to bind to specific gene loci [52]. On the other hand, histone deacetylases (HDACs) remove acetyl groups which lead to tighter winding of DNA and decreased transcription of tumor suppression genes such as genes involved in cell cycle arrest, apoptosis, cell proliferation and survival, and angiogenesis [19,52]. Furthermore, global loss of monoacetylation of histone H4 is a hallmark of human tumor cells [53]. Another key epigenetic regulator is DNA methyltransferase (DNMT). DNMTs transfer a methyl group from methyl donor S-adenosyl-L-methionine to the cytosine in DNA and the methylation status regulates gene silencing, transcriptional activation, and posttranslational regulation [54]. DNMTs mutations are associated with cancer development. Hypermethylation can result in uncontrolled growth because of silenced growth regulatory genes and DNA methylation inhibitors that can activate silenced tumor suppressors genes have been actively tested as candidate anticancer drugs [55]. In our study, we found decreased HDAC2 and HDAC8 expression, inhibited HDACs activity, decreased DNMT3b expression, and decreased DNMTs activity, indicating that inulin dietary treatment induced protective epigenetic changes in mammary tumor cells in mice.

Since HDACs and DNMTs closely regulate the proliferation and survival of tumor cells, we further studied the expression of key factors involved in the Akt/PI3K pathway and cell cycle arrest. The AKT/PI3K pathway promotes metabolism, proliferation, and survival of tumor cells, and plays an important role in endocrine resistance in BC [56,57]. The protein expression of downstream factors such as NF-kB and Bcl-xL were also measured. NF-kB, a proinflammatory transcription factor, facilitates hormone-independent and invasive BC tumor development. Inhibiting NF-kB sensitizes cancer cells to apoptotic effects of chemotherapies and radiotherapies and is correlated with higher disease-free survival of BC patients [58]. Bcl-xL is an apoptosis inhibitor that was found to be correlated with metastatic potential. The silencing of Bcl-xL significantly decreased migration in the MDA-MB-231 cell line [59]. The cell cycle is another downstream factor that can be influenced by the Akt-PI3K pathway. Deregulation of the cell cycle enables cancer cells to proliferate unlimitedly. Anticancer agents that targets cell cycle pathway have been explored, including CDK4/6 inhibitors and PLK4 inhibitors [60]. In the present study, we found that the protein expression of p-PI3K, NF-kB, and Bcl-xL decreased in 8% IN or 15% IN groups. CDK6 decreased in the 10% IN group and Cyclin D1 decreased in both the 10% and 15% IN groups. These findings suggest that inulin dietary treatments greatly suppress mammary tumorigenesis through downregulating factors involved in cell proliferation and cell survival.

The mechanism of inulin dietary treatment on BC tumor development was hypothesized to be mediated in part by propionic acid in the plasma. Therefore, to verify the anticancer effect of propionic acid in vitro, human BC cell lines were treated with various concentrations of sodium propionate (SP) for testing the cytotoxicity. Previous study showed that 5 mM and 10 mM SP can significantly reduce the cell viability of ER-negative BC cell line JIMT-1 and ER-positive BC cell line MCF-7 under 1, 2, or 3 days treatment and the anticancer mechanism was mediated by decreased proliferating cell nuclear antigen and cell cycle arrest [31]. In the present study, the time- and dose-dependent cytotoxicity effect of SP was studied. Importantly, the SP decreased cell viability of not only ER-positive cell lines, but also ER-negative BC cell lines MDA-MB-231 and MDA-MB-157, while the immortalized noncancerous MCF10A human mammary epithelial control cells were not significantly affected by SP treatment. Furthermore, the epigenetics modification of SP on human BC cell lines was evaluated by enzymatic activities of key epigenetic regulators HDACs and DNMTs. Decreased HDACs and DNMTs activities in cancerous cell lines suggest that SP induced protective epigenetic modification in vitro. To further study the in vivo anticancer effect of sodium propionate, an ER-negative BC mouse model was treated with sodium propionate in the drinking water. Previous studies showed 1 *w*/*v*% SP in drinking water ameliorated body weight loss and colonic damage by reducing inflammation and oxidative stress in colitis mice [30]. In another study, 100 mg/kg SP orally administered to mice reduced tumor growth in glioblastoma by promoting the PPAR-gamma signaling in the apoptosis and autophagy pathways [61]. The anticancer effect of SP has also been studied in the BC models. SP at 20 mg/mL (2 *w*/*v*%) in drinking water suppressed tumor growth by downregulating STAT3 and activating p38 in JIMT-1 and MCF-7 xenograft mouse models [31]. In the present study, 1 *w*/*v*% SP in drinking water decreased tumor incidence and decreased tumor weight and latency although not significantly, suggesting SP had potential anticancer effects on the ER-negative transgenic mouse model. A longer treatment period and higher concentrations of SP, for example 2 *w*/*v*% SP, in drinking water may further enhance this efficacy in future studies given the encouraging results of these initial studies. The molecular mechanism of the anticancer effect of 1 *w*/*v*% sodium propionate treatment on the SV40 mouse model was investigated by Western blotting and measuring enzymatic activities. The protein expression of enzymatic activities of key epigenetic modifiers HDACs and DNMTs decreased. Therefore, we show that SP can induce protective epigenetics modification not only in vitro but also in vivo.

**Figure 12:**
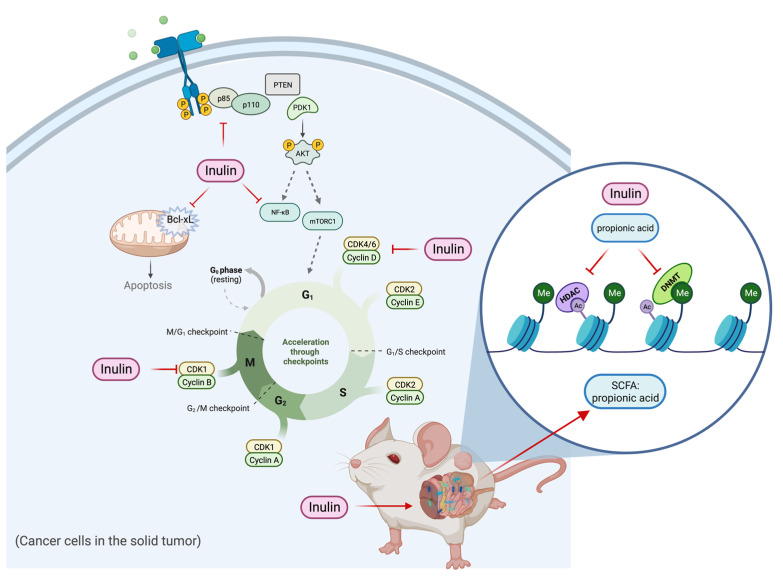
Hypothetical schematic for mechanism of action of tumor reduction in inulin-fed mice. The figure shows a proposed mechanism of the inulin-supplemented diet in cancer prevention. The inulin-supplemented diet shaped gut microbial composition and increased the plasma propionic acid level in an ER-negative BC mouse model. The gut microbial composition changes were associated with protective epigenetic regulation and decreased cell proliferation partially mediated by increased level of plasma propionic acid.

In summary, the inulin-supplemented dietary treatment can profoundly suppress ER-negative mammary tumorigenesis in the transgenic mouse model via shaping gut microbial composition and increasing plasma propionic acid level that was shown to regulate HDACs and DNMTs expression and their enzymatic activities and regulate cell proliferation and cell survival pathways. The hypothetical schematic of a proposed mechanism is shown (Figure 12). This study may provide implications into new perspectives of inulin dietary treatment. As an HDACs inhibitor with antiproliferation properties, inulin might serve beyond being a health supplement for weight loss and improving bowel movement. Application of dietary inulin to prevent or treat ER-negative BC, which is generally untreatable with more conventional anticancer approaches, could be an exciting new avenue for controlling this deadly disease. Further research into the anticancer effect of dietary fibers and prebiotics is needed to better understand the relationship between gut microbial composition and protective epigenetic changes in the host.

## 4. Materials and Methods

### 4.1. Animal Experiments

#### 4.1.1. Mouse Model

We used breast cancer transgenic mouse models FVB/N-Tg(MMTV-Erbb2)NK1Mul/J (Her2/neu) and C3(1)-SV40 Tag (FVB-Tg(C3-1-Tag)cJeg/Jeg) (C3(1)-TAg). The Her2/neu mouse model overexpresses activated oncogene *Erbb2* and spontaneously develops ER-negative mammary cancer tumors at 20 weeks with an average latency at 25 weeks [19,62]. C3(1)-TAg mouse model expresses Tag gene and spontaneously develops mammary cancer tumors resembling Ductal Carcinoma in situ (DCIS) at 15 weeks of age [63,64]. Animal models were purchased from the Jackson Laboratory (Bar Harbor, ME, USA). Protocols were reviewed and approved by the Institutional Animal Care and Use Committee and animal studies were monitored by the Animal Resources Program of the University of Alabama at Birmingham (UAB). Mice were housed in the Animal Resource Facility of the UAB and were maintained in conditions: 12-h dark/12-h light cycle, 24 ± 2 °C, 40–60% humidity.

#### 4.1.2. Dietary Treatment

Inulin-supplemented diet pellets AIN-93M were purchased from the Teklad/Envigo (Indianapolis, IN, USA). 8% (wt:wt),10% (wt:wt), and 15% (wt:wt) inulin was added to the AIN-93M diet. AIN-93M was used as the control diet to the inulin-supplemented diets. The details of dietary ingredients were provided in the Supplemental materials (TD. 94048, TD. 200417, TD. 200418, TD.200419) (Figures S1–S4). Forty 5-wk old female Her2/neu mice were randomly divided into four groups (10 mice/group) and each group was fed control diet or inulin-supplemented diet: (1) control group: AIN-93M; (2) 8% inulin-supplemented AIN-93M; (3) 10% inulin-supplemented AIN-93M; (4) 15% inulin-supplemented AIN-93M. Treatment started at 4 weeks and terminated at 29 weeks. Food was delivered to mice ad libitum. Food intake was measured at 15, 20, and 25 weeks of age and body weight was measured at 12, 16, 20, 24, and 28 weeks of age (Figure S5).

A 1% (wt:wt) sodium propionate (SP) purchased from Millipore Sigma (P1880) (Burlington, MA, USA) was added to the drinking water. Forty 10-week old female C3(1)-TAg mice were randomly divided into two groups (20 mice/group): (1) control group; (2) 1% SP-added drinking water. Treatment started at 10 weeks and terminated at 22 weeks. Water was replaced every three days and delivered to mice ad libitum. Water intake was measured at 15, 18, and 21weeks of age and body weight was measured at 12, 14, 16, 18, 20, and 22 weeks of age (Figure S6).

#### 4.1.3. Mammary Tumor Evaluation and Sample Collection

Tumor volume, incidence, and latency of transgenic mouse models Her2/neu and C3(1)-TAg were measured and recorded weekly. Tumor volume was calculated using the formula: length (cm) × width (${\mathrm{cm}}^{2}$) × 0.523 [19,65]. Treatments were terminated when all mice in the control group developed tumors with average volume of 1.0 cm^3^. At the termination point, tumors were collected, weighed, and stored in liquid nitrogen. Blood samples were collected from the retroorbital sinus of mice to tubes containing EDTA. Plasma is prepared by centrifuging blood samples at 1500 rcf for 10 min at 4 °C. Plasma samples were stored at −80 °C for further analysis.

#### 4.2. 16S rRNA Sequencing and Gut Microbiome Analysis

Fecal samples were collected from the Her2/neu mouse model. Samples (7–8/group) were collected from the control group, 8% inulin group, and 15% inulin group at two times points: (1) 15 weeks, before tumor onset; (2) 25 weeks, after tumor onset. Genomic DNA was extracted using Fecal DNA Isolation Kit, Zymo Research (Irvine, CA, USA) according to the manufacturer’s instructions. The isolated DNA was quantified using microspectrophotometer (ThermoFisher, Waltham, MA, USA) [66]. Using PCR and unique barcoded primers, an amplicon library was constructed from isolated DNA to amplify the V4 region of the 16S rRNA gene. The primers were as follows (Eurofind Genomics, Inc., Huntsville, AL, USA): Forward V4: 5′-AATGATACGGCGACCACCGAGATCTACACTATGGTAATTGTGTGCCAGCMGCCGCGGTAA-3′ and Reverse V4: 5′CAAGAGAAGACGGCATACGAGATNNNNNNAGTCAGTCAGCCGGACTACHVGGGTWTCTAAT-3′. PCR products were quantified by PICO green dsDNA Reagent. Quantified PCR products were purified by QIAquick Gel Extraction Kit (Qiagen, Germantown, MD, USA). The sequencing of purified PCR products was performed by the NextGen Sequencing Illumina MiSeq platform [66,67]. The raw FASTQ files were de-multiplexed, assessed for quality control using FastQC, and analyzed using the Quantitative Insight into Microbial Ecology (QIIME) data analysis package [68]. Uclust clustering program was used to group samples into amplicon sequence variant (ASV) with 97% similarity and to evaluate phylum level changes. Multiple sequence alignment of ASV was generated by PyNAST [69]. Alpha diversity was calculated with the Shannon and Simpson diversity index. Beta diversity was calculated with Bray Curtis and weighed Unifrac methods to quantify the dissimilarity between treatment groups [67,70].

### 4.3. Plasma Short-Chain Fatty Acid Measurement by LC-MS/MS

Samples (4–5/group) were used for measuring plasma short-chain fatty acids levels. Plasma samples were collected from the Her2/neu mouse model at the termination time of the inulin-supplemented dietary treatment and stored in an −80 °C freezer. Then, 1.0 mM acetic acid, 0.1 mM of all measured acids, 200 mM ^13^C_6_ labeled 3-nitrophenyl hydrazine (3-NPH, IsoSciences) (Ambler, PA, USA), and 120 mM N-(3-dimethylaminopropyl)-N’-ethylcarbodiimide hydrochloride were added to EtOH/water to prepare the stable-isotope internal standard solution. Volumetrically dissolving SCFAs in 50% ethanol was performed to make a mixture of C2–C8 short-chain fatty acids as calibration standards. Samples were prepared using the Standard Operating Procedure for SCFA analysis based on previous studies [71]. After derivatization, samples were analyzed by LC-MS/MS. Briefly, Waters Acquity LC was used for UPLC separation of the SCFAs. Electrospray ionization in negative mode was used for a Xevo TQ-S mass spectrometer in the Multiple Reaction Monitoring mode. The standard curve was run once at the beginning of the run queue and once at the end of the sample run. Raw data, peak integration, and linear regression with 1/x2 weighting for calibration curves were analyzed in Skyline v21.1.9 (www.skyline.ms) (https://skyline.ms/project/home/software/Skyline/begin.view 1 October 2022). Lower limit of quantitation and upper limit of quantitation were used to determine the lowest and highest points.

### 4.4. Western Blotting Analysis

For tissue samples, three randomly selected tumor samples/group of Her2/neu mouse model were used. Protein extracts of mammary tumors were prepared by homogenizing 50 mg tumor followed by protein extraction using T-PER Tissue Protein Extraction Reagent (Thermo Fisher Scientific) (Waltham, MA, USA) according to the manufacturer’s protocol. Briefly, each tumor sample was homogenized in 1 mL of T-PER with protease/phosphatase inhibitor cocktail (100×) (Cell Signaling) (Danvers, MA, USA). Supernatant was collected after the sample was centrifuged at 10,000 rcf for 5 min at 4 °C. For cell lines, protein extract of whole cell lysate was prepared. Briefly, RIPA buffer with protease/phosphatase inhibitor cocktail (100×) was added to cells. Supernatant was collected after each sample was centrifuged at 16,000 rcf for 20 min at 4 °C. The Bradford Assay was used to measure the concentration of protein samples. Denatured protein samples were separated by 4–15% NuPAGE Tris-HCl gels (Invitrogen) (Carlsbad, CA, USA) by electrophoresis and transferred to nitrocellulose membranes. Membranes were probed with antibodies, including: Akt, p-PI3k, hdac1, hdac2, hdac3, hdac8, dnmt1, dnmt3a, dnmt3b, nf-Κb, bcl-xL, bax, Cyclin D, Cylin E, cdk61, cdk6, (Cell Signaling) (Danvers, MA, USA). The list of antibodies was attached in the Supplementary Material Table S8. Β-actin was probed as loading control on the same membranes. Clarity Max Western ECL Blotting Substrates (Bio-Rad) (Hercules, CA, USA) was added to membranes to visualize immunoreactive bands using ChemiDoc XRS+ System (Bio Rad) (Hercules, CA, USA). Protein expression was quantified by Image J software Version 1.53.

### 4.5. Cell Culture and Treatment

Four cancerous BC cell lines were used: two human triple-negative breast cancer (TNBC) cell lines, MDA-MD-231 and MDA-MB-157, and two Erα-positive BC cell lines, MCF-7 and T-47D. Noncancerous human mammary epithelial cell line MCF10A was used as a control for the cell viability assay. All five cell lines were purchased from American Type Culture Collection (ATCC) (Manassas, VA, USA). Cancerous BC cell lines were grown in Dulbecco’s Modified Eagle’s Medium (DMEM, Corning) (New York, NY, USA) supplemented with 10% fetal bovine serum (FBS, Corning) (New York, NY, USA) and 1% penicillin/streptomycin (Corning) (New York, NY, USA). MCF10A was grown in DMEM/F12 medium (Corning) (New York, NY, USA) supplemented with 5% fetal bovine serum and 1% penicillin/streptomycin (Corning) (New York, NY, USA), 0.5 mg/mL hydrocortisone and 100 ng/mL cholera toxin and 10 ug/mL insulin (Millipore Sigma) (Burlington, MA, USA), and 20 ng/mL EGF (PeproTech) (Rocky Hill, NJ, USA). All cell lines were maintained in an incubator under 37 °C, 5% CO_2_, and humidified conditions. Cells were treated with sodium propionate (SP) (≥99.0, BioReagent, suitable for insect cell culture, Millipore Sigma) (Burlington, MA, USA). Fresh 4 mM SP culture medium was replaced every 24 h during the treatment. After 72 h of treatment, cells were collected for further analysis.

### 4.6. MTT Analysis

Cell viability of SP treatment on four cancerous BC cell lines MDA-MB-231, MDA-MB-157, MCF-7 and T-47D and one noncancerous cell line MCF10A was analyzed. Cells were seeded in 96-well plates and treated with 0, 0.5, 1, 2, 3, and 4 mM of SP for 24 h, 48 h, and 72 h. Fresh medium was replaced every 24 h. Culture medium was discarded after treatment. One mg/mL 3-(4,5-dimethylthiazol-2-yl)-2,5-diphenyltetrazolium bromide (MTT) was added to each well on the 96-well plates. Plates were incubated for 3 h in a 37 °C incubator. MTT was discarded and 100 μL DMSO was added to all plates. After shaking plates on a rocking platform for 15 min at room temperature, the absorbance was measured at 570 nm using a microplate reader (Epoch model, Biotek) (Shoreline, WA, USA).

### 4.7. Histone Acetyltransferase, DNA Methyltransferase, and Histone Acetyltransferase Assays

For in vivo, three randomly selected tumor samples/group of Her2/neu mouse model were used. For in vitro, after cells were collected after 72 h of 4 Mm SP treatment. Nuclear protein of tumor samples and cells were both extracted using EpiQuik Nuclear Extraction Kit (EpiGentek) (Farmingdale, NY, USA) according to the manufacture’s protocol. Histone acetyltransferase activity was measured using EpiQuik HDAC Activity/Inhibition Assay Kit (Colorimetric) (EpiGentek) (Farmingdale, NY, USA). DNA methyltransferase activity was measured using EpiQuik DNMT Activity/Inhibition Assay ELISA Easy Kit (Colorimetric) (Epigentek) (Farmingdale, NY, USA). Histone acetyltransferase was measured using EpiQuik HAT Activity/Inhibition Assay Kit (Epigentek) (Farmingdale, NY, USA). Manufacturer’s protocols were followed for all assays.

### 4.8. Statistical Analysis

Statistical analysis of phenotypic data was performed by GraphPad Prism (version 9.5.0). The two-group comparisons were analyzed by two-tailed Student’s *t*-test. The comparisons of three or more groups were analyzed by one-way ANOVA with Tukey’s post-hoc test. Tumor incidence was calculated by chi-square testing. Error bars were presented as mean ± standard error of the mean and statistically significant results were considered at *p* < 0.05. For gut microbiome analysis, permutational multivariate analysis of variance (PERMANOVA) was used for analyzing the Bray-Curtis test and weighted-UniFrac test. PCoA group comparison and Kruskal Wallis test were performed to investigate taxonomic abundance between groups. The statistical significance was adjusted with a false discovery rate (FDR) at 5%. All graphs were created by GraphPad Prism (version 9.5.0) or BioRender (BioRender.com).

## Data Availability

Data are contained within the article or Supplementary Materials.

## Supplementary Materials

The supporting information can be downloaded at: https://www.mdpi.com/article/10.3390/ijms24109015/s1.
