# Supplementary material for: Dietary Supplementation of Inulin Contributes to the Prevention of Estrogen Receptor-Negative Mammary Cancer by Alteration of Gut Microbial Communities and Epigenetic Regulations
Source: Int J Mol Sci. 2023 May 19;24(10):9015. doi: 10.3390/ijms24109015 (PMC10218871; doi:10.3390/ijms24109015)
Supplement: Supplementary file 1 [file ijms-24-09015-s001.zip › Fig. S1 94048 AIN93M.pdf]

Teklad Custom Diet

TD.94048

AIN-93M Purified Diet

Formula

Formula	g/Kg
Casein	**140.0**
L-Cystine	**1.8**
Corn Starch	**465.692**
Maltodextrin	**155.0**
Sucrose	**100.0**
Soybean Oil	**40.0**
Cellulose	**50.0**
Mineral Mix, AIN-93M-MX (94049)	**35.0**
Vitamin Mix, AIN-93-VX (94047)	**10.0**
Choline Bitartrate	**2.5**
TBHQ, antioxidant	**0.008**

Footnote

Reference: J. Nutr. 123:1939-1951, 1993. Formulated for the maintenance of adult, non-reproducing rodents.

Selected Nutrient Information[1]

	% by weight	% kcal from
**Protein**	12.4	13.7
**Carbohydrate**	68.3	75.9
**Fat**	4.1	10.3

Kcal/g 3.6

[1] Values are calculated from ingredient analysis or manufacturer data

Speak With A Nutritionist

+ **(800) 483·5523**
+ **askanutritionist@envigo.com**

Teklad diets are designed & manufactured for research purposes only.

Key Features

+ **Purified Diet**
+ **American Institute of Nutrition**
+ **Maintenance**
+ **Commonly Used**

Key Planning Information

+ *Products are made fresh to order*
+ Store product at 4°C or lower
+ Use within 6 months (applicable to most diets)
+ Box labeled with product name, manufacturing date, and lot number
+ Replace diet at minimum once per week

  More frequent replacement may be advised
+ Lead time:
  - 2 weeks non-irradiated
  - 4 weeks irradiated

Product Specific Information

+ 1/2" Pellet or Powder (free flowing)
+ Minimum order 3 Kg
+ Irradiation not advised
  - Contact a nutritionist for recommendations

Options (fees will apply)

+ Rush order (pending availability)
+ Irradiation (see Product Specific Information)
+ Vacuum packaging (1 and 2 Kg)

Contact Us

Obtain pricing · Check order status

+ **teklad@envigo.com**
+ **(800) 483·5523**

International Inquiry *(outside USA or Canada)*

+ **askanutritionist@envigo.com**

Place Your Order (USA & Canada)

Please Choose One

+ **envigo.com/teklad-orders**
+ **tekladorders@envigo.com**
+ **(800) 483·5523**
+ **(608) 277·2066** *facsimile*

RJR

© 2015 Envigo

09/22/15
