# Supplementary material for: Dietary Supplementation of Inulin Contributes to the Prevention of Estrogen Receptor-Negative Mammary Cancer by Alteration of Gut Microbial Communities and Epigenetic Regulations
Source: Int J Mol Sci. 2023 May 19;24(10):9015. doi: 10.3390/ijms24109015 (PMC10218871; doi:10.3390/ijms24109015)
Supplement: Supplementary file 1 [file ijms-24-09015-s001.zip › Fig. S3 200418 10% IN.pdf]

Teklad Custom Diet

TD.200418

10% Inulin Diet (93M, O)

Formula

Formula	g/Kg
Casein	140.0
L-Cystine	1.8
Corn Starch	365.592
Maltodextrin	155.0
Sucrose	100.0
Soybean Oil	40.0
Cellulose	50.0
Mineral Mix, AIN-93M-MX (94049)	35.0
Vitamin Mix, AIN-93-VX (94047)	10.0
Choline Bitartrate	2.5
TBHQ, antioxidant	0.008
Inulin	100.0
Orange Food Color	0.1

Footnote

Modification of AIN-93M/TD.94048 to include 10% inulin and orange food coloring.

Selected Nutrient Information[1]

	% by weight	% kcal from
**Protein**	12.4	14.7
**Carbohydrate**	62.3	74.2
**Fat**	4.1	11.1
**Kcal/g**	**3.4**	

[1] Values are calculated from ingredient analysis or manufacturer data

Speak With A Nutritionist

+ **(800) 483·5523**
+ **askanutritionist@envigo.com**

Teklad diets are designed & manufactured for research purposes only.

Key Features

+ **Purified Diet**
+ **AIN-93M Modification**
+ **Inulin**
+ **Color Coded Orange**

Key Planning Information

+ *Products are made fresh to order*
+ Store product at 4°C or lower
+ Use within 6 months (applicable to most diets)
+ Box labeled with product name, manufacturing date, and lot number
+ Replace diet at minimum once per week
  More frequent replacement may be advised
+ Lead time:
  · 2 weeks non-irradiated
  · 4 weeks irradiated

Product Specific Information

+ 1/2" Pellet or Powder (free flowing)
+ Minimum order 3 Kg
+ Irradiation not advised
  · Contact a nutritionist for recommendations

Options (fees will apply)

+ Rush order (pending availability)
+ Irradiation (see Product Specific Information)
+ Vacuum packaging (1 and 2 Kg)

Contact Us

Obtain pricing · Check order status

+ **teklad@envigo.com**
+ **(800) 483·5523**

International Inquiry *(outside USA or Canada)*

+ **askanutritionist@envigo.com**

Place Your Order (USA & Canada)

Please Choose One

+ **www.envigo.com/teklad-orders**
+ **tekladorders@envigo.com**
+ **(800) 483·5523**
+ **(608) 277·2066** *facsimile*

#N/A

© 2015 Envigo

06/12/20
