# Supplementary material for: Dietary Supplementation of Inulin Contributes to the Prevention of Estrogen Receptor-Negative Mammary Cancer by Alteration of Gut Microbial Communities and Epigenetic Regulations
Source: Int J Mol Sci. 2023 May 19;24(10):9015. doi: 10.3390/ijms24109015 (PMC10218871; doi:10.3390/ijms24109015)
Supplement: Supplementary file 1 [file ijms-24-09015-s001.zip › Fig. S5 and S6 and Table S8.pdf]

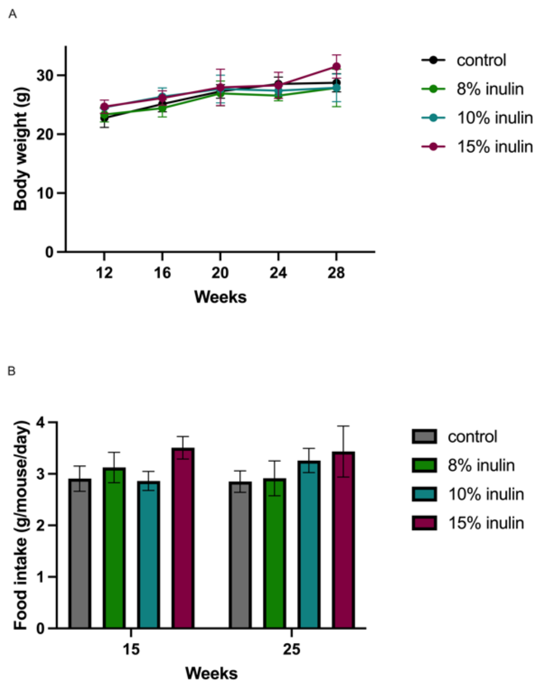

Supplemental Figure S5. The body weight and food intake of Her2/neu mouse model. (a) Mouse body weight and (b) food intake of Her2/neu mice in the control, 8% IN, 10% IN and 15% IN groups. Mean with SEM error bars. Mouse weight was recorded at 12, 16, 20, 24, 28 wks of age. Food intake was recorded at 15 and 25 wks of age. Control, AIN93M diet; 8% IN, 8 w/w% inulin supplemented in AIN93M diet; 10% IN, 10 w/w% inulin supplemented in AIN93M diet; 15% IN, 15 w/w% inulin supplemented in AIN93M diet.

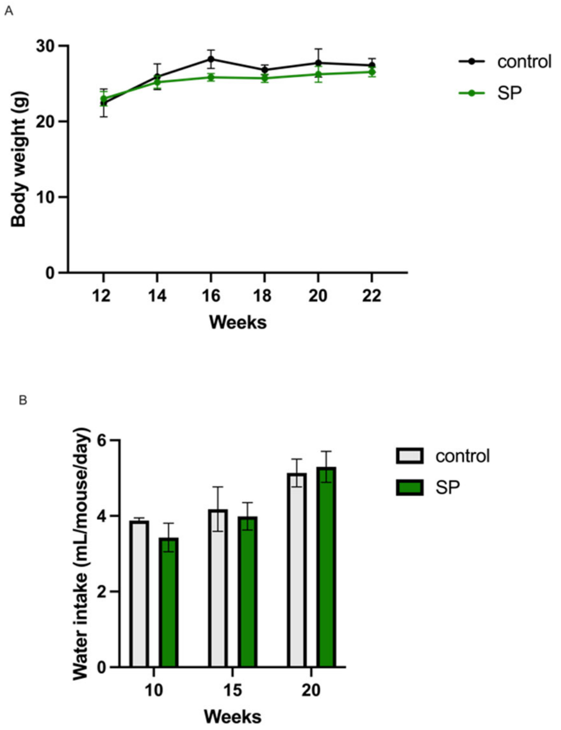

Supplemental Figure S6. The body weight and water intake of C3(1)-TAg mouse model. (a) Mouse body weight and (b) water intake of SV40 mice in the control and 1 w/v% sodium propionate groups. Mean with SEM error bars. Mouse weight was recorded at 12, 14, 16, 18, 10, 22 wks of age. Food intake was recorded at 10, 15 and 20 wks of age. Control, water; SP, water supplemented with 1 w/v% sodium propionate powder.

Antibody	MW/kDa	Species	Source	Catalogue no.	Dilution ratio
CDK1	34	Rabbit	Cell Signaling Technology	28439	1:1000
CDK6	34	Mouse	Cell Signaling Technology	3136	1:1000
Cyclin D1	36	Rabbit	Cell Signaling Technology	2978	1:1000
Cyclin E1	48	Rabbit	Cell Signaling Technology	20808	1:1000
AKT	62	Mouse	Santa Cruz	81434	1:1000
p-PI3K p85/p55	60, 85	Rabbit	Cell Signaling	4228	1:1000
NF-kB	64	Mouse	Santa Cruz	8008	1:1000
BCL-XL	30	Rabbit	Cell Signaling Technology	2764	1:1000
DNMT3A	130	Rabbit	Cell Signaling Technology	3598	1:1000
DNMT3B	97	Mouse	Santa Cruz	sc-81252	1:1000
DNMT1	200	Rabbit	Cell Signaling	5032	1:1000
HDAC1	60	Mouse	Cell Signaling Technology	2062	1:1000
HDAC2	60	Rabbit	Cell Signaling	57156	1:1000
HDAC3	49	Rabbit	Cell Signaling	85057	1:1000
HDAC6	160	Rabbit	Cell Signaling	7612	1:1000
HDAC8	45	Rabbit	Cell Signaling	66042	1:1000
ACTB	45	Rabbit	Cell Signaling Technology	4970	1:2000

Supplemental Table S8 Information of primary antibodies for western blot in this study
